# Oleanolic Acid Suppresses Aerobic Glycolysis in Cancer Cells by Switching Pyruvate Kinase Type M Isoforms

**DOI:** 10.1371/journal.pone.0091606

**Published:** 2014-03-13

**Authors:** Jia Liu, Ning Wu, Leina Ma, Ming Liu, Ge Liu, Yuyan Zhang, Xiukun Lin

**Affiliations:** 1 Institutes of Oceanology, Chinese Academy of Sciences, Qingdao, China; 2 Graduate School, University of Chinese Academy of Sciences, Beijing, China; 3 Department of Pharmacology, Capital Medical University, Beijing, China; 4 Department of Molecular Biology, School of Medicine and Pharmacy, Ocean University of China, Qingdao, China; University of South Alabama, United States of America

## Abstract

Warburg effect, one of the hallmarks for cancer cells, is characterized by metabolic switch from mitochondrial oxidative phosphorylation to aerobic glycolysis. In recent years, increased expression level of pyruvate kinase M2 (PKM2) has been found to be the culprit of enhanced aerobic glycolysis in cancer cells. However, there is no agent inhibiting aerobic glycolysis by targeting PKM2. In this study, we found that Oleanolic acid (OA) induced a switch from PKM2 to PKM1, and consistently, abrogated Warburg effect in cancer cells. Suppression of aerobic glycolysis by OA is mediated by PKM2/PKM1 switch. Furthermore, mTOR signaling was found to be inactivated in OA-treated cancer cells, and mTOR inhibition is required for the effect of OA on PKM2/PKM1 switch. Decreased expression of c-Myc-dependent hnRNPA1 and hnRNPA1 was responsible for OA-induced switch between PKM isoforms. Collectively, we identified that OA is an antitumor compound that suppresses aerobic glycolysis in cancer cells and there is potential that PKM2 may be developed as an important target in aerobic glycolysis pathway for developing novel anticancer agents.

## Introduction

Aerobic glycolysis, also known as Warburg effect, has been established as a hallmark of cancer cells [1]. Almost all types of cancer cells change their metabolism by increasing glycolysis and suppressing mitochondrial oxidative phosphorylation, even under normoxic conditions (therefore defined as aerobic glycolysis) [2]. Many glycolytic intermediates are indispensible for the synthesis of molecules that is essential for cellular structures and functions, such as nucleotides, amino acids and lipids. Thus, highly proliferating cancer cells meet their requirements for cellular building materials by switching their metabolism from oxidative phophorylation to glycolysis, although ATP production is less efficient in glycolytic process.

*PKM* gene encodes two protein kinases, PKM1 and PKM2, and these kinases are also responsible for the conversion of phosphoenolpyruvate (PEP) to pyruvate which can be used for lactic acid production or enterring mitochondrial oxidative phosphorylation. PKM1 and PKM2 are generated by exclusive mRNA splicing (exon 9 for PKM1 and exon 10 for PKM2) [3]. http://en.wikipedia.org/wiki/PKM2 - cite_note-Corcoran-5PKM1 is catalytically more active than PKM2. PKM2 is expressed in all cells with a high rate of nucleic acid synthesis [4]. Cancer cells utilize PKM2 to accumulate the intermediates for the synthesis of nucleic acid and protein and maintain aerobic glycolysis [5]. Increasing PKM2 activity or switching mRNA splicing from PKM2 to PKM1 is able to suppress the Warburg effect, and consequently, compromise tumor growth [6]. Therefore, PKM2 is believed to a promising target in the field of cancer therapy. However, the compounds that can increase the ratio of PKM1 to PKM2 have not been found.

Oleanolic acids (OA) is distributed widely in many plants, and it has been well documented that OA displays anti-tumor activity to a range of human cancer cells. Previous study in our laboratory has shown that treatment of human pancreatic pan-28 cancer cells leading to apoptosis via mitochondrial mediated apoptotic pathway [7]. Another study also reveals that OA can inhibit metastasis on glioma cells [8]. However, the target of OA has not been well identified. In the present study, we found that OA can suppress aerobic glycolysis by suppressing PKM2 expression and affected *PKM* mRNA splicing through mTOR/c-Myc/hnRNP signaling.

## Methods and Materials

### Cell Culture and Chemical Compounds

Human prostate carcinoma cell line, PC-3, and human breast cancer cell line, MCF-7, were purchased from American Type Culture Collection C (ATCC). PC-3 cells were cultured in RPMI-1640 supplemented with 10% fetal bovine serum (FBS; GIBCO-BRL) at 37°C under a humidified 5% CO_2_ condition. MCF-7 cells were cultured in Dulbecco’s modified Eagle’s medium (DMEM).

Oleanolic acid (OA) was purchased from Sigma Aldrich (O5504, St. Louis, MO). OA was prepared in DMSO at the concentration of 10 mg/ml as stock solution.

### Cell Transfection

Plasmids, including pWZL Neo Myr Flag PKM2 and pMXs-hcMYC were obtained from Addgene (Cambridge, MA). pcDNA Flag GFP (Flag-GFP) was preserved in our laboratory and was used as control. Cell transfection was performed using Lipofectamine 2000 (Invitrogen, CA) according to the manufacturer’s instructions. Briefly, PC-3 or MCF-7 cells were plated onto a 96-well plate. When the cells are cultured to approximately 85% confluence, the media were replaced with OPTI-MEM. Then, 3 µg plasmids and 30 µl lipofectamine reagent was mixed in a tube containing 1500 µl OPTI-MEM by vigorous vortexing. After incubation for 15 min, the mixture was added to the cell cultures and incubated for certain times.

### Immunoblotting Assays

Cells were harvested by centrifugation at 1000 g for 10 min and lyzed with M-PER Mammalian Protein Extraction Reagent (Thermo Scientific, IL). Protein concentrations were determined using the BCA protein assay kit (Thermo Scientific, FL). The total protein was separated with electrophoresis with 10–12% polyacrylamide gel and transferred onto 0.45 µm nitrocellulose membranes. Membranes were incubated in blocking solution (PBS, 0.1% Tween-20, and 5% nonfat dry milk powder) for 2 hr at room temperature, and then, incubated with primary antibodies (1∶1000). After incubation overnight, the membranes were washed with PBS containing 0.1% Tween-20 for three times, and then, were incubated with horseradish peroxidase-conjugated secondary antibody (IgG goat anti-rabbit or anti-mouse; 1∶2000; Bio-Rad, CA) for 1 hr at room temperature. The protein bands were visualized with SuperSignal West Dura Extended Duration Substrate (Thermo Scientific, IL). The intensity of the blots were quantified using ImageJ software.

The primary antibodies used in our study were as follows: PKM1 (#7067, Cell Signaling Technology, Danvers, MA), PKM2 (#4053, Cell Signaling Technology, Danvers, MA), β-tubulin (#2146, Cell Signaling Technology, Danvers, MA), Phospho-mTOR (Ser2448) (#2971, Cell Signaling Technology, Danvers, MA); mTOR (#2983, Cell Signaling Technology, Danvers, MA); c-Myc, (sc-40, Santa Cruz Biotechnology, Dallas, Texas); hnRNP A1 (#8443, Cell Signaling Technology, Danvers, MA); hnRNP A2 (#9304, Cell Signaling Technology, Danvers, MA).

### Immunofluorescence Analysis

PC-3 and MCF-7 cells (5×10^4^/well) were plated onto 24-well plates, and then, OA (100 µg/ml) was added. After 6 hr incubation, the cells were fixed with 4% Polyoxymethylene for 0.5 hr, followed by incubation with PKM2 antibody (1∶1000) for 2 hr. The corresponding secondary antibody was added and incubated for another 1 hr to visualize PKM2. 4′,6′-diamidino-2-phenylindole (DAPI) was used to stain the nuclei. The fluorescence was detected under a confocal microscope (CLS-2SS, Thorlabs).

### Metabolic Assays

Assays of O_2_ content, pyruvate kinase (PK) activity, glucose uptake, lactate production, and cellular respiration were used to study the changes in metabolic switch in cancer cells treated with OA. The Pyruvate Kinase (PK) Assay Kit (ab83432, Abcam) and OX-500 O_2_ Microsensor (Unisense, Denmark) were used to determine PK activity and O_2_ content, respectively, in PC-3 and MCF-7 cell treated with OA, following the manufacturer’s instructions.

To measure the glucose uptake, PC-3 and MCF-7 were seeded at a density of 5×10^3^ cells/well in 96-well plates. After incubation overnight, the cells were transfected with certain plasmids or treated with MHY1485. Then, the cells were treated with 50 or 100 µg/ml OA, respectively. After incubation for 6 or 12 hr, the culture media were harvested by centrifugation at 3000 g for 15 min. The quantity of glucose consumption of the tested cancer cells was detected using Glucose Uptake Colorimetric Assay Kit I (#K676, BioVision, Milpitas, CA) according to the manufacturer’s instructions. The absorption density (OD) at 412 nm was read using a BioRad680 microplate reader (Bio-Rad, Hercules, CA).

The production of lactic acid was determined using Lactate Colorimetric Assay Kit II (#K627, BioVision, Milpitas, CA) as the manufacturer’s protocols. Briefly, PC-3 and MCF-7 cells were treated with pWZL Neo Myr Flag PKM2, pMXs-hcMYC and pcDNA Flag GFP for 72 hr or MHY1485 for 1 hr, respectively. OA was added at the indicated concentrations and incubated for the indicated times. Then the culture media were collected by centrifugation at 3000 g for 15 min, mixed with 45 µl of Lactate Assay Buffer. The absorbance at 450 nm was read using a BioRad680 microplate reader (Bio-Rad, Hercules, CA).

The oxygen consumption was examined using Oxygen Consumption Rate Assay Kit (#600800, Cayman Chemical Company, Ann Arbor, MI) following the manufacturer’s instruction. VICTOR X3 Multilabel Plate Reader (Perkin Elmer) was used to detect the OD value at 650 nm. The oxygen consumption rate was shown as lifetime versus time (µs/hr).

### Colony Formation Assay

PC-3 or MCF-7 cells were plated onto a 6-well plate, and transfected with plasmids, including pWZL Neo Myr Flag PKM2 and pcDNA Flag GFP. After incubation for certain times, the cells were trypsinized, suspended in DMEM containing 10% FBS, plated onto a bottom layer containing 0.6% agar and covered with a top layer containing 0.6% agar. The cells were cultured in a 6-well plate and the media were changed weekly. After incubation for 10 days, crystal violet was added and the number of colonies was counted and analyzed with ImageJ software.

### Statistical Analysis

The experiments except immunoblot assays were performed for at least three times. All values were expressed as means ± SD, and compared at a given time point by unpaired, two-tailed t test. Data were considered to be statistically significant when p<0.05 (*) and p<0.01 (**).

## Results

### OA Inhibits Aerobic Glycolysis in Cancer Cells

**Figure 1:**
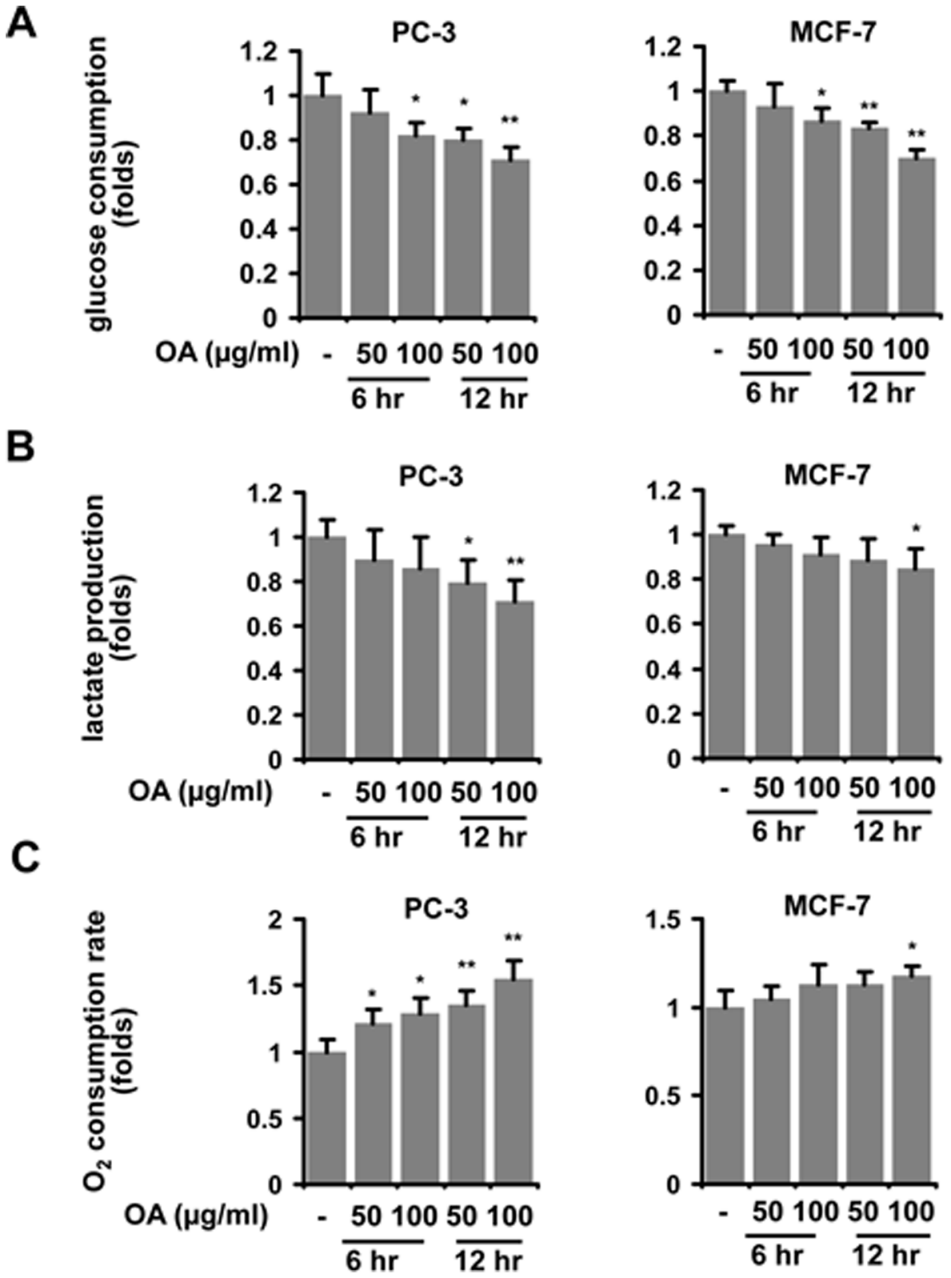
OA suppresses the aerobic glycolysis in cancer cells. Glucose consumption (**A**), lactic acid production (**B**) and oxygen consumption (**C**) were assessed in PC-3 and MCF-7 cells treated with 50 or 100 µg/ml OA for 6 or 12 hr respectively. The details of methodology have been described in the section of Materials and Methods. The ratio of each group to control was presented here. The bars showed the average values of three independent experiments (Mean ± SD). *, P<0.05; **, P<0.01.

Aerobic glycolysis is characterized by increased glucose uptake and lactate production, and compromised oxygen consumption in cancer cells with high availability to oxygen. Therefore, we measured the effect of OA on these activities of cancer cell. Reduced glucose consumption was found in several types of cancer cells when OA was added to the cultures **(** **Fig. 1A** **)**. The production of lactic acid in these cancer cells was also found to be decreased in cells treated with OA **(** **Fig. 1B** **)**. Furthermore, OA enhanced the consumption of oxygen in cancer cells **(** **Fig. 1C** **)**. The above data showed that OA was able to induce the metabolic alteration of cancer cells by suppressing aerobic glycolysis.

In addition, we compared O_2_ content in cancer cells with or without OA treatment. The data showed that OA has no influence on the concentration of oxygen in the cell culture, thereby excluding the possibility that OA lead to metabolic alteration by affecting normic conditions **(Figure S1)**.

### OA Induces the Switch from PKM2 to PKM1 and Increase PK Activity in Cancer Cells

**Figure 2:**
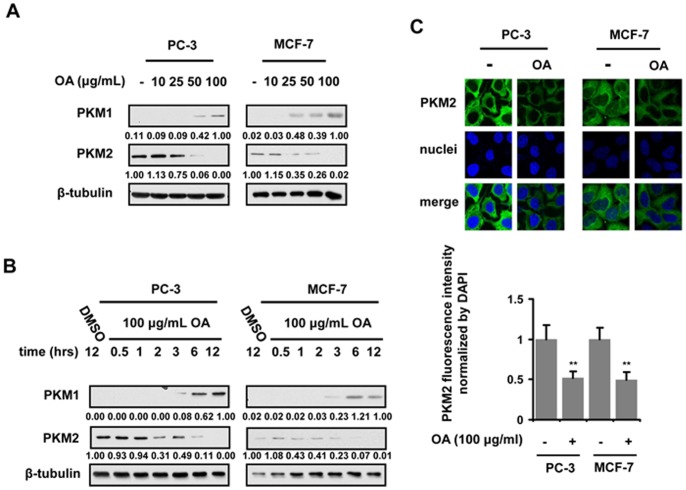
OA treatment affects the expression profile of PKM isoforms in cancer cells in a dose- and time-dependent manner. PKM1 and PKM2 expression level was evaluated by immunoblotting assays in PC-3 and MCF-7 cells treated with 10, 25, 50,100 µg/ml OA respectively for 12 hr (**A**) or 100 µg/ml OA for 0.5, 1, 2, 3, 6, 12 hr respectively (**B**). β-tubulin was used as endogenous references. (**C**) The PKM2 levels (Green) were in PC-3 and MCF-7 cells treated with 100 µg/ml OA for 12 hr respectively, as determined by immunfluorescent staining. The nuclei (Blue) were stained with DAPI. The merged pictures were shown in the bottom of the panel. The fluorescence intensity was quantified with ImageJ software. The ratio of each group to control was presented here. The bars represented the average values of 5 randomly selected experiments.

Pyruvate kinase muscle isozyme 2 (PKM2), whose enzymatic activity is less active than PKM1, is responsible for the conversion of phosphopyruvate into pyruvate in the glycolytic process. PKM2, especially in its dimeric form, accumulates glycolytic intermediates and channels them into the biosynthesis of nucleotide and amino acids, thereby contributing to the growth of cells. PKM2 has been found to be highly expressed in all proliferating cells, such as tumor cells [4]. To identify the mechanisms that OA affected aerobic glycolysis, we subsequently evaluated the changes in the expression level of PKM1 and PKM2 in cancer cells treated with OA. The immunoblot analysis revealed that OA was able to decrease PKM2 abundance, and increase PKM1 expression, in both PC-3 and MCF-7cancer cell lines, in a dose- and time-dependent manner **(** **Fig. 2A and 2B** **)**. The reduction in PKM2 expression was also found in Immunofluorescent staining experiments. The treatment of cancer cells with OA down-regulated the PKM2 expression significantly in both PC-3 and MCF-7 cells **(** **Fig. 2C** **)**. Furthermore, PK activity was shown to be elevated in OA-treated cancer cells, along with the switch from PKM2 to PKM1 **(Figure S2A and S2B)**.

### The Increase in PK Activity Caused by PKM2/PKM1 Switch Mediates OA Suppression of Aerobic Glycolysis

**Figure 3:**
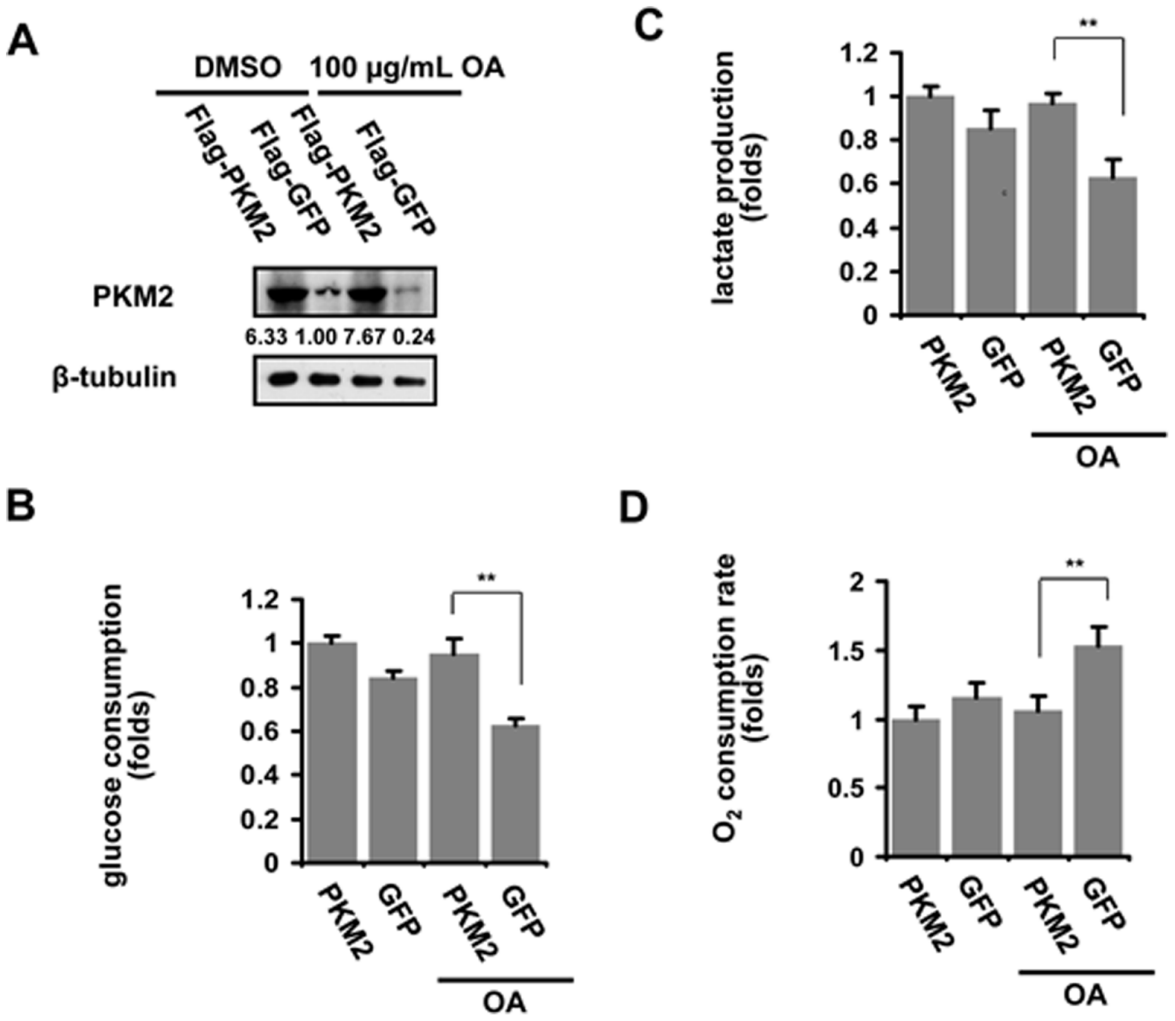
PKM2 overexpression abolishes the effect of OA on aerobic glycolysis in cancer cells. (**A**) OA-treated PC-3 cells were transfected with pWZL Neo Myr Flag PKM2 (Flag-PKM2) or control vector (Flag-GFP). Immunoblot assays were performed to determine the expression of PKM2 protein. β-tubulin was used as endogenous references. Glucose consumption (**B**), lactic acid production (**C**) and oxygen consumption (**D**) were assessed in PC-3 cells treated with 100 µg/ml OA for 12 hr. The details of methodology was described in the section of Materials and Methods. The ratio of each group to control was presented here. The bars showed the average values of three independent experiments (Mean ± SD). *, P<0.05; **, P<0.01.

Based on the finding that the switch from PKM2 to PKM1 occurs in cancer cells treated with OA, we subsequently investigated the role of increaed PK activity in OA suppression of aerobic glycolysis. PC-3 cells were transfected with PKM2- or GFP-expressing vector prior to OA stimulation **(** **Fig. 3A** **)**. OA-induced increase in PK activity was rescued by PKM2 overexpression in PC-3 cells **(Figure S3)**. The results also showed that the glucose consumption, the lactate production as well as the oxygen consumption were all restored in cells overexpressing PKM2 **(** **Fig. 3B, 3C and 3D** **)**. The results suggest that increased PK activity due to PKM2/PKM1 switch is required for the inhibitory effect of OA on aerobic glycolysis in cancer cells.

### OA Suppression of mTOR Mediates the Alteration in the Expression Profiles of PKM Isoforms and Metabolic Switch in Cancer Cells

**Figure 4:**
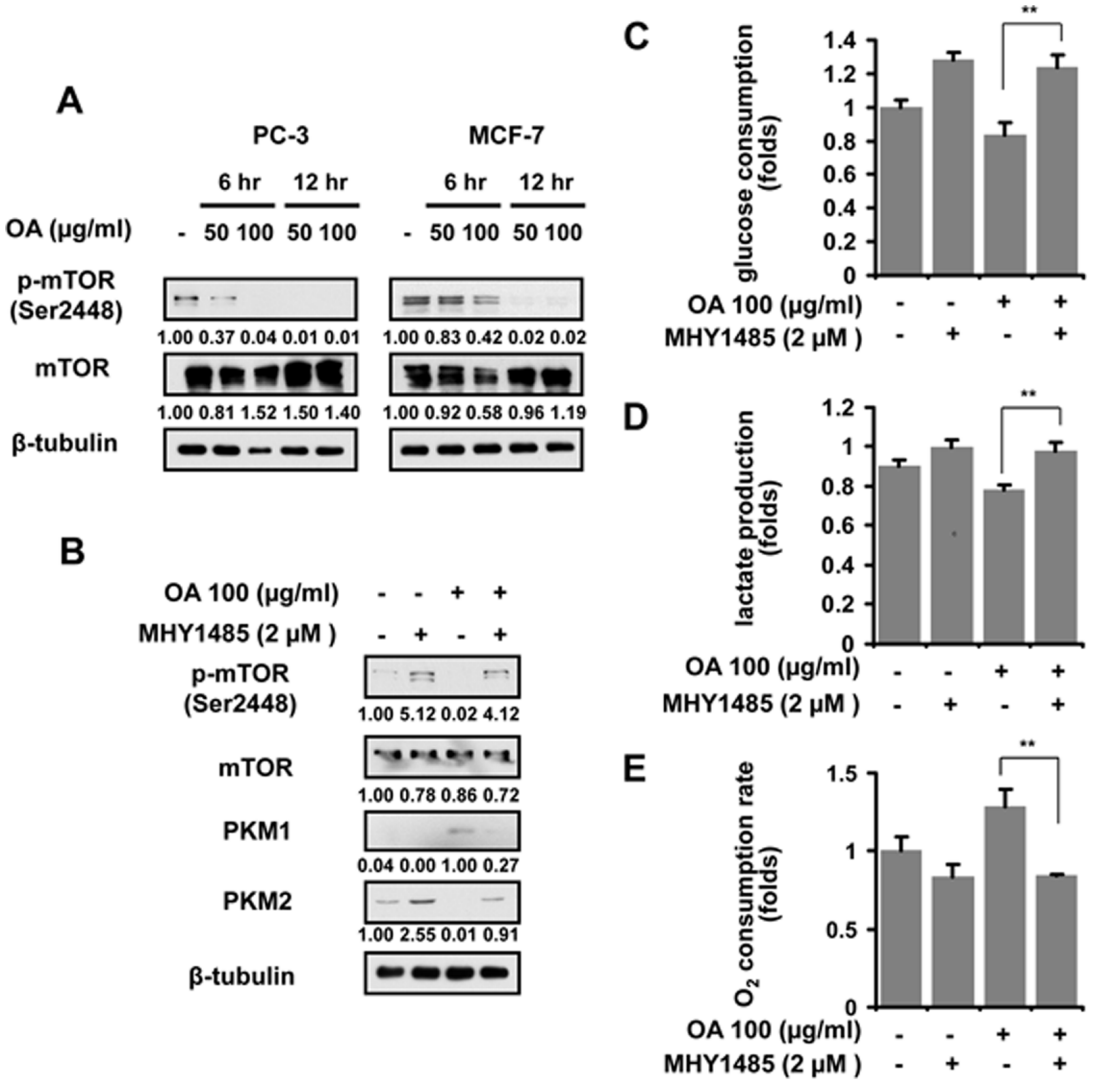
mTOR suppression is required for OA-induced metabolic switch. (**A**) The levels of phosphorylated mTOR was examined in PC-3 and MCF-7 cells treated with 50 or 100 µg/ml OA for 6 or 12 hr respectively as determined by immunoblot assays. β-tubulin was used as endogenous references. (**B**) Immunoblot analysis of PKM1 and PKM2 expression was performed in PC-3 cells incubated with OA (100 µg/ml) or/and a mTOR activator MHY1485 (2 µM). Glucose consumption (**C**), lactic acid production (**D**) and oxygen consumption (**E**) were detected in PC-3 cells treated with OA (100 µg/ml) and/or MHY1485 (2 µM) for 12 hr. The ratio of each group to control was presented here. The bars showed the average values of three independent experiments (Mean ± SD). *, P<0.05; **, P<0.01.

We were interested in how OA induced the decrease in the switch from PKM2 to PKM1 in cancer cells. mTOR signaling has been demonstrated to participate in the switch of PKM isoforms in cancer cells [9]. Therefore, we detected the changes in active status of mTOR in OA-treated cancer cells. Immunoblotting analysis revealed that mTOR activation was diminished when OA was added to the culture of cancer cells **(** **Fig. 4A** **)**. Reactivating mTOR by MHY1485 protected cancer cells from OA-mediated decline in PKM2 expression and increase in PKM1 levels **(** **Fig. 4B** **)**. Our further study showed that the glucose consumption, the lactate production and the oxygen consumption rate are also restored in cells treated with MHY1485 **(** **Fig. 4C, 4D and 4E** **)**. The result reveals that mTOR is responsible for the OA induced the switch of PKM isoforms and metabolism.

### OA-induced mTOR Suppression Induces the Switch of PKM Isoforms by Inhibiting c-Myc-dependent hnRNPA1 and hnRNPA2 Expression

**Figure 5:**
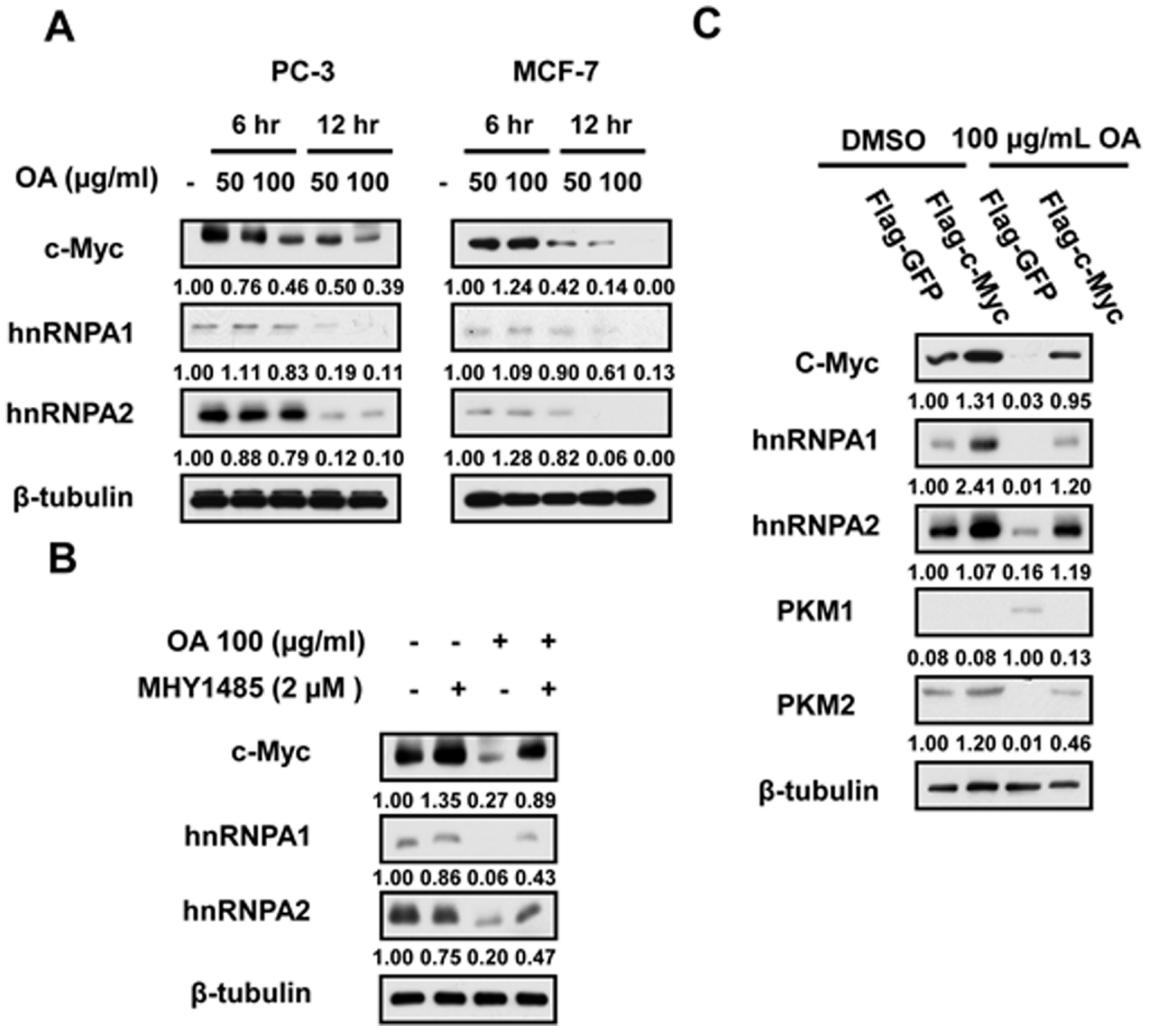
The switch from PKM2 to PKM1 by OA results from the decrease in Myc, hnRNPA1 and hnRNPA2 expression. (**A**) The expression of c-Myc, hnRNPA1 and hnRNPA2 was determined by immunoblot analysis in PC-3 and MCF-7 cells treated with 50 or 100 µg/ml OA for 6 or 12 hr respectively. (**B**) The expressionof c-Myc, hnRNPA1 and hnRNPA2 expression was determined by immunoblot analysis in PC-3 cells treated with OA (100 µg/ml) or/and MHY1485 (2 µM) respectively. (**C**) The level of c-Myc, hnRNPA1, hnRNPA2, PKM1 and PKM2 was determined by immunoblot assays in PC-3 cells treated with plasmids pMXs-hcMYC (Flag-cMyc) in the presence or absence of OA. β-tubulin was used as endogenous references.

mTOR has been demonstrated to elevate PKM2 level by stimulating c-myc-dependent hnRNPA1 and hnRNPA2 expression [9], both of which are responsible for the exclusive splicing of PKM mRNA [3]. We subsequently determined the influence of OA on the expression of hnRNPA1 and hnRNPA2. The results showed that OA can reduce the expression level of c-Myc, as well as its target gene hnRNPA1 and hnRNPA2 expression **(** **Fig. 5A** **)**. mTOR activation mediated by MHY1485 was able to restore the expression of c-Myc, hnRNPA1, and hnRNPA2 in OA-treated cancer cells **(** **Fig. 5B** **)**. Furthermore, transfection of OA-treated cancer cells with plasmids expressing c-Myc abrogated the effect of OA on the change in PKM1 and PKM2 expressions **(** **Fig. 5C** **)**. The results suggest that OA can induce PKM2/PKM1 switch via c-Myc-dependent hnRNPA1 and hnRNPA2 mTOR signal pathway.

### Reduced Aerobic Glycolysis Partially Accounts for the Anti-tumor Activity of OA

**Figure 6:**
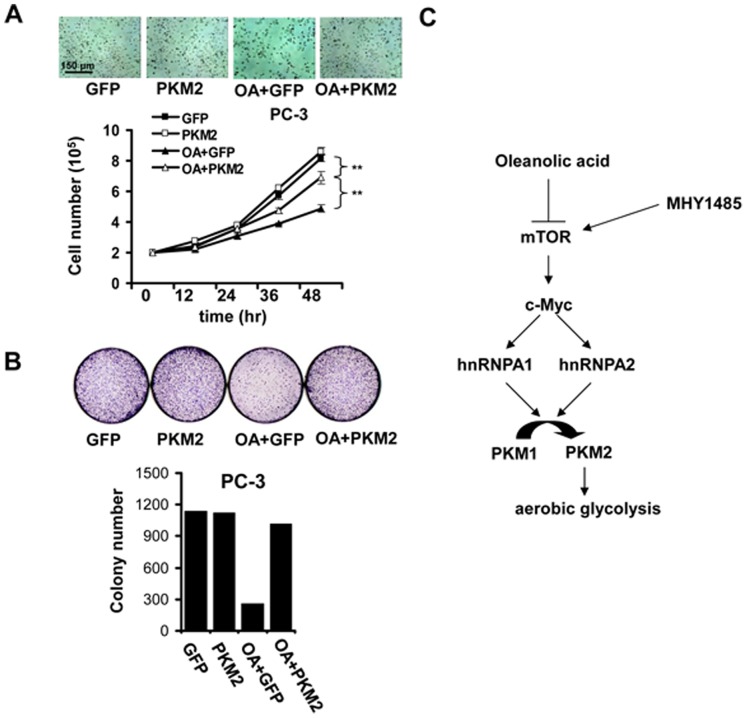
OA suppression of aerobic glycolysis contributes to its anti-tumor activity. (**A**) PC-3 cells was counted 12, 24, 36 and 48 hr after the treatment of OA (100 µg/ml) or/and MHY1485 (2 µM) using hemacytometry. The average values of three independent experiments were shown as Mean ± SD. **, P<0.01. The representative figure was shown for each group. (**B**) Cells were treated with OA (100 µg/ml) or/and MHY1485 (2 µM) for 10 days, the number of colonies of PC-3 cells were counted and analyzed using ImageJ software. The representative figure was shown for each group. (**C**) OA suppresses the activation of mTOR in cancer cells. This inhibitory effect on mTOR signaling, in turn, abrogated c-Myc/hnRNPA1/hnRNPA2-dependent PKM2 expression. Consequently, the aerobic glycolysis was inhibited in cancer cells treated with OA.

To address the contribution of impaired aerobic glycolysis to tumor suppressor activity of OA on cancer cells, we detected the changes in malignant phenotypes of OA-stimulated cancer cells which still underwent aerobic glycolysis due to PKM2 overexpression. OA was found to reduce the growth of PC-3 cells **(** **Fig. 6A**). Also, the number of colonies formed in PC-3 cells was also decreased under the treatment of OA (OA+GFP group) **(** **Fig. 6B** **)**. However, PKM2 restoration significantly abolished the effect of OA on cancer cells**,** evidenced by the increase in the growth rate and the number of formed colonies in PC-3 cells co-treated with OA and PKM2-expressing vectors (OA+PKM2 group) **(** **Fig. 6A and 6B** **)**.

In summary, OA suppresses the activation of mTOR signaling in cancer cells. In turn, the inhibitory effect of OA on mTOR pathway leads to the switch from PKM2 to PKM1 expression, which is mediated by c-Myc/hnRNPA1/hnRNPA2 pathway. Eventually, aerobic glycolysis was suppressed in OA-treated cancer cells **(** **Fig. 6C** **)**.

## Discussion

OA, a natural compound widely distributed in plants, has been documented to possess a variety of bioactivities including anti-tumor property [10]. The mechanisms of OA’s anti-tumor activity involve apoptosis induction, cell cycle arrest and metastasis inhibition [7], [8]. However, the effect of OA on the metabolic pathway in cancer cells is still unclear. In this study, we found that OA was able to suppress the Warburg effect in cancer cells, evidenced by increased oxygen consumption, reduced lactate production and lower glucose uptake **(** **Fig. 1A–1C** **)**. To our knowledge, this is the first time to reveal that OA affects the metabolic switch of cancer cells. Our finding may contribute to the understanding of OA action on cancer cells and help to the development of OA and its derivatives with more potent efficacy for clinical application.

Aerobic glycolysis is believed to be a novel, important and effective target in cancer chemotherapy. Increasing evidences indicate that aerobic glycolysis plays a critical role in the onset and progression of malignant diseases. Several compounds have been reported to suppress the growth of tumor cells by affecting aerobic glycolysis[11]–[15]. For instances, the extract from Spatholobus suberectus was shown to inhibit the activity of lactate dehydrogenase A (LDHA), thereby suppressing the aerobic glycolysis in breast cancer cells [13]. An anti-inflammatory compound, curcumin, has recently been demonstrated to reverse Warburg effect caused by Tumor Necrosis Factor (TNF) stimulation in breast cancer cells [14]. Our study confirmed that OA also possesses the inhibitive activity on aerobic glycolysis. The study increases the understanding of anticancer mechanism of OA.

Many studies have revealed that PKM2 plays a key role in aerobic glycolysis in cancer cells [3]. In the present study, we found that the PKM2 expression can be inhibited in cells treated with OA in a dose- and time-dependent manner **(** **Fig. 2A–2C** **)**. More importantly, increased PK activity, due to PKM2/PKM1 switch, mediated the effect of OA on aerobic glycolysis in cancer cells, since reducing PK activity by overexpressing PKM2 level in cancer cells abolished the OA-induced metabolic switch **(** **Fig. 3A–3D** **)**. OA is therefore identified as the first compound reducing PKM2 abundance, and exerts its effect on metabolism in cancer cells through a new target. Therefore, we provided initial evidences that targeting PKM2 may be a novel strategy to develop cancer agents, and OA may be a lead compound for developing anti-cancer drugs targeting PKM2.

Aberrant activation of mTOR signaling is closely implicated in a variety of cancers [16]. mTOR activation promotes the resistance of cancer cells to apoptosis induced by both cytokines and chemotherapeutic agents, accelerates the proliferation of a wide range of cancer cells, and facilitates the metastasis [16]. Most of the current mTOR inhibitors suppress the growth of cancer cells primarily by inducing apoptosis and cell cycle arrest, or impairing metastasis [17]–[19]. In this study, we found that OA reduces the expression level of phosphorylated mTOR in cancer cells **(** **Fig. 4A** **)** associated with its inhibitory activity on aerobic glycolysis. Recently, mTOR signaling has been proven to modulate the metabolic switch in cancer cells and this effect is mediated by the change in PKM2 expression [14], [20], [21]. Furthermore, mTOR/PKM2 pathway is responsible for, at least, liver tumorigenesis [22]. These new findings suggest that targeting metabolism may be a novel strategy of mTOR inhibitor to suppress the growth of cancers.

Collectively, we proved evidence that OA was able to switch metabolic patterns from aerobic glycolysis to oxidative phosphorylation through affecting mTOR/c-Myc/PKM2 pathway in cancer cells. Considering its low toxicity to normal tissues, OA and its analoges may be a promising candidate agent for developing anticancer agents targeting metabolism. Our studies contribute to a better understanding on the mechanism of OA’s antitumor property, and also the study provides primary evidence that PKM2 can be selected as a critical therapeutic target in aerobic glycolysis pathway in developing novel anticancer agents.

## Supporting Information

Figure S1 **OA failed to affect the content of oxygen in cell culture.** PC-3 and MCF-7 cells were treated with or without OA for 12 hr. The O_2_ content was detected with at the indicated time points. The average values of three independent experiments were shown as Mean ± SD.
(PPT)

Figure S2 **OA incubation induced the elevation in PK activity in cancer cells.** (**A**) PC-3 and MCF-7 cells were treated with OA at the indicated doses and PK activity was determined 12 hr after OA treatment. The average values of three independent experiments were shown as Mean ± SD. *, P<0.05, **, P<0.01. (**B**) The activity of PK was also assessed in cancer cells exposed with 100 µg/ml OA at the indicated time points. The average values of three independent experiments were shown as Mean ± SD. *, P<0.05, **, P<0.01.
(PPT)

Figure S3 **PKM2 overexpression rescue the elevation in PK activity induced by PKM2/PKM1 switch.** PK activity was evaluated in OA-treated PC-3 cells transfected with PKM2- or GFP-expressing vector, 12 hr after the treatment of 100 µg/ml OA. The average values of three independent experiments were shown as Mean ± SD. *, P<0.05.
(PPT)
