# Supplementary figures and images for: Oleanolic Acid Suppresses Aerobic Glycolysis in Cancer Cells by Switching Pyruvate Kinase Type M Isoforms
Source: PLoS One. 2014 Mar 13;9(3):e91606. doi: 10.1371/journal.pone.0091606 (PMC3953484; doi:10.1371/journal.pone.0091606)

## Slide 1
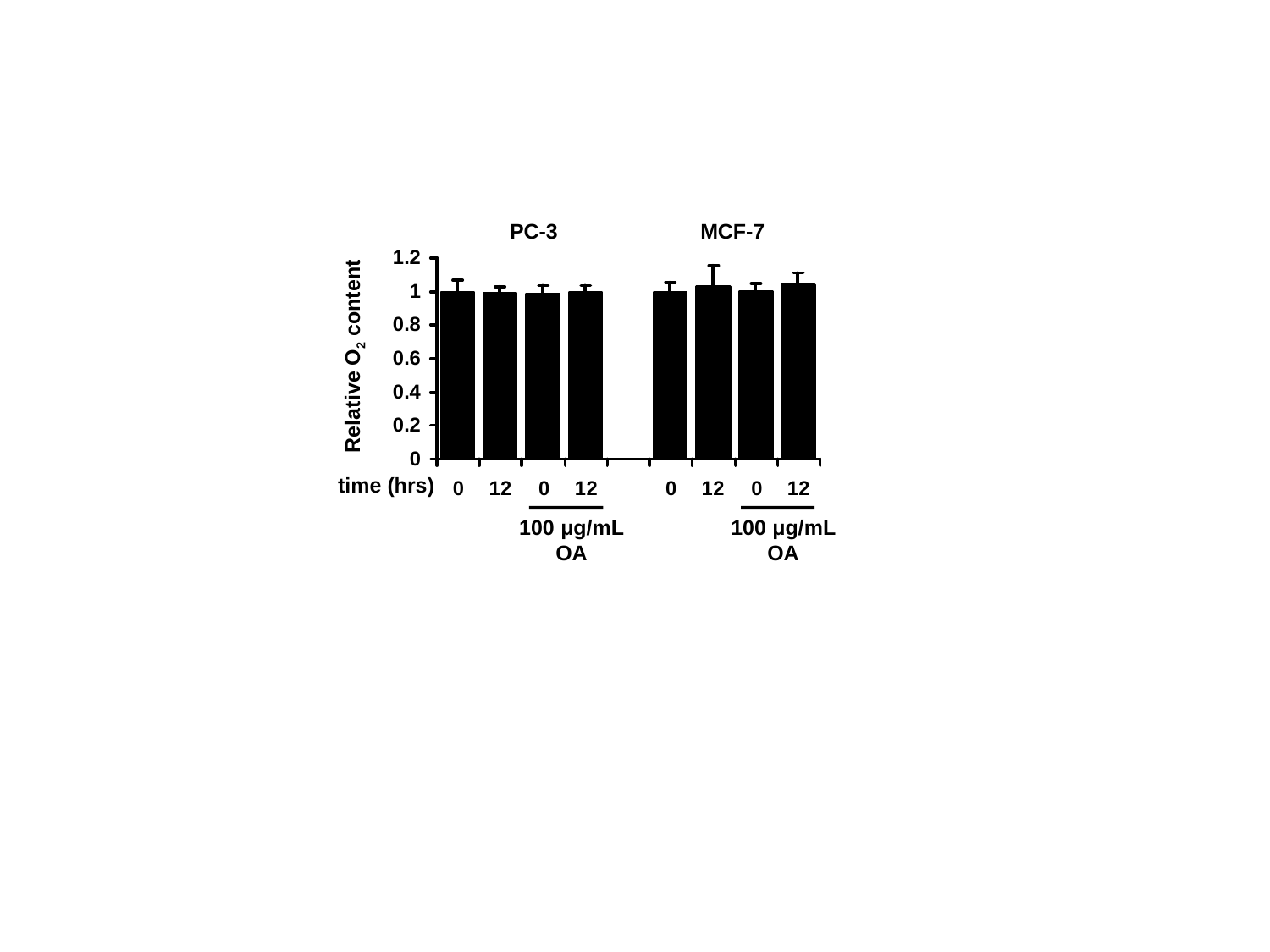

PC-3
MCF-7
Relative O2 content
time (hrs)
100 μg/mL OA
100 μg/mL OA

Supplement: Figure S1 — OA failed to affect the content of oxygen in cell culture. PC-3 and MCF-7 cells were treated with or without OA for 12 hr. The O2 content was detected with at the indicated time points. The average values of three independent experiments were shown as Mean ± SD. (PPT) [file pone.0091606.s001.ppt]

## Slide 1
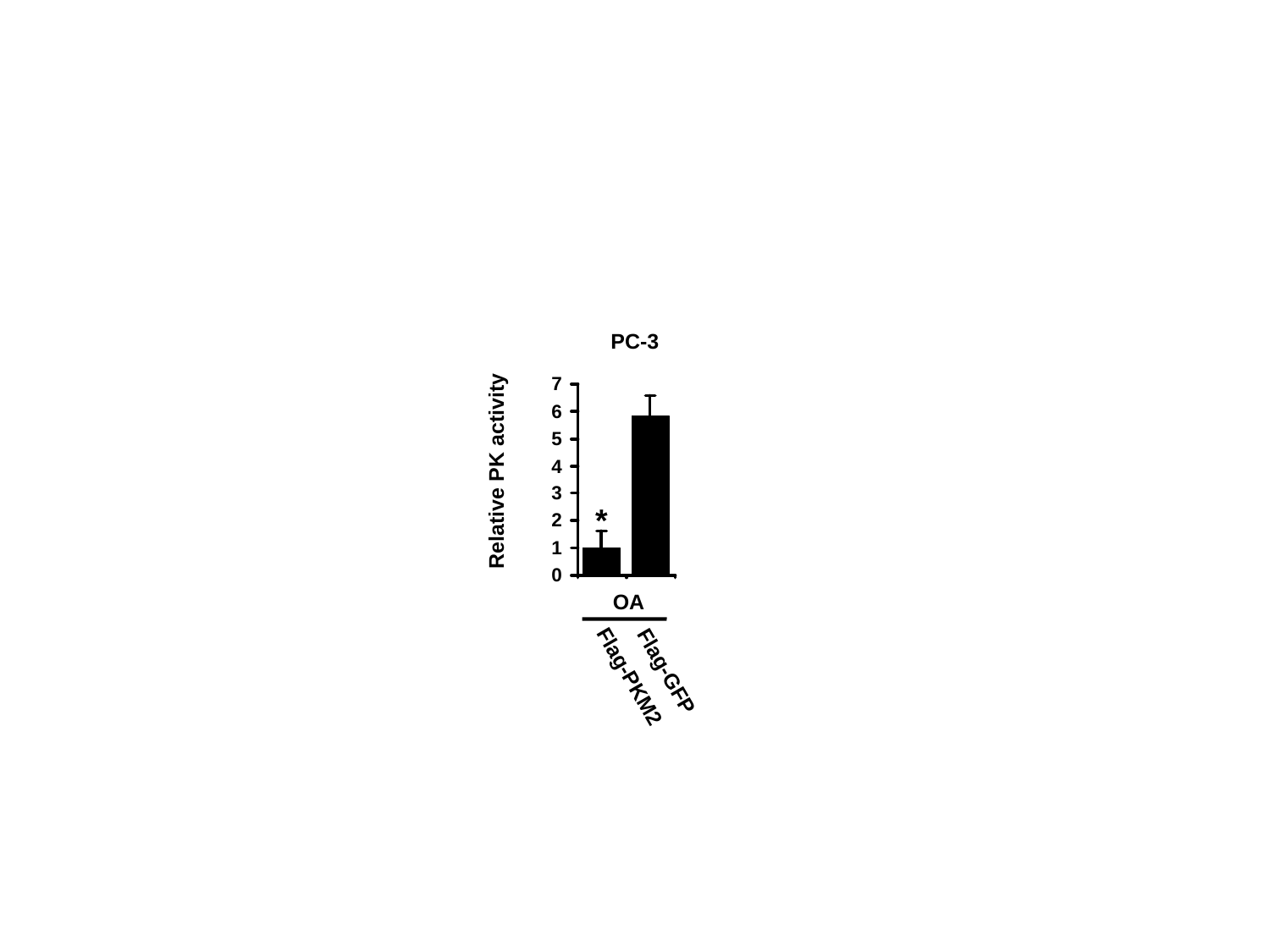

PC-3
Relative PK activity
*
OA
Flag-GFP
Flag-PKM2

Supplement: Figure S3 — PKM2 overexpression rescue the elevation in PK activity induced by PKM2/PKM1 switch. PK activity was evaluated in OA-treated PC-3 cells transfected with PKM2- or GFP-expressing vector, 12 hr after the treatment of 100 µg/ml OA. The average values of three independent experiments were shown as Mean ± SD. *, P<0.05. (PPT) [file pone.0091606.s003.ppt]
