# Supplementary material for: Oleanolic Acid Suppresses Aerobic Glycolysis in Cancer Cells by Switching Pyruvate Kinase Type M Isoforms
Source: PLoS One. 2014 Mar 13;9(3):e91606. doi: 10.1371/journal.pone.0091606 (PMC3953484; doi:10.1371/journal.pone.0091606)
Supplement: Figure S2 — OA incubation induced the elevation in PK activity in cancer cells. (A) PC-3 and MCF-7 cells were treated with OA at the indicated doses and PK activity was determined 12 hr after OA treatment. The average values of three independent experiments were shown as Mean ± SD. *, P<0.05, **, P<0.01. (B) The activity of PK was also assessed in cancer cells exposed with 100 µg/ml OA at the indicated time points. The average values of three independent experiments were shown as Mean ± SD. *, P<0.05, **, P<0.01. (PPT) [file pone.0091606.s002.ppt]

## Slide 1
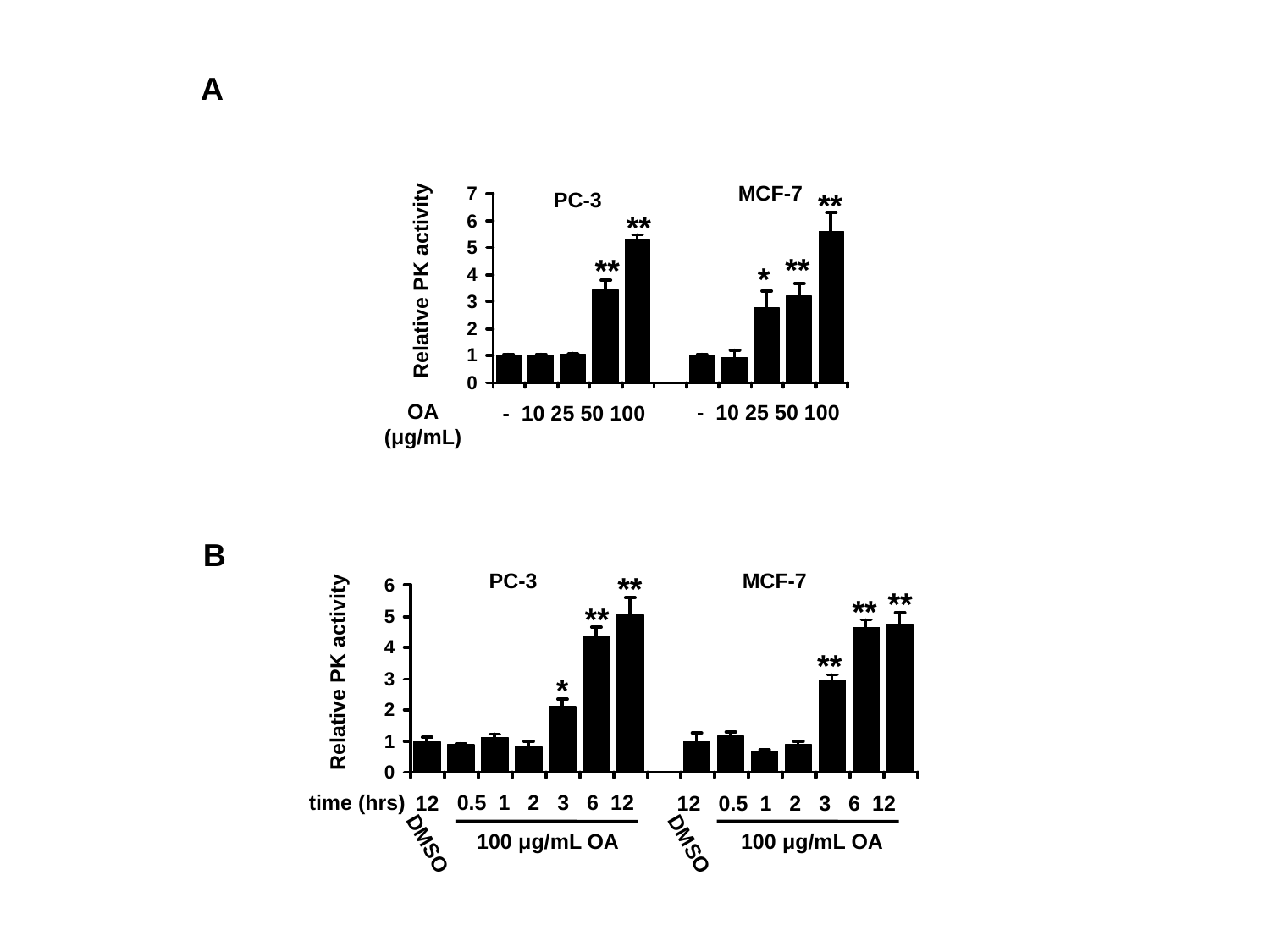

A
MCF-7
**
PC-3
**
**
**
*
Relative PK activity
OA (μg/mL)
- 10 25 50 100
- 10 25 50 100
B
PC-3
MCF-7
**
**
**
**
**
Relative PK activity
*
time (hrs)
0.5 1 2 3 6 12
12
0.5 1 2 3 6 12
12
100 μg/mL OA
100 μg/mL OA
DMSO
DMSO
